# Movement and perceptual strategies to intercept virtual sound sources

**DOI:** 10.3389/fnins.2015.00149

**Published:** 2015-05-06

**Authors:** Naeem Komeilipoor, Matthew W. M. Rodger, Paola Cesari, Cathy M. Craig

**Affiliations:** ^1^Department of Neurological and Movement Sciences, University of VeronaVerona, Italy; ^2^MOVE Research Institute, VU University AmsterdamAmsterdam, Netherlands; ^3^School of Psychology, Queen's University BelfastBelfast, UK

**Keywords:** perception-action coupling, sound interception, tau, interaural time difference, temporal control of movement, moving sound source

## Abstract

To intercept a moving object, one needs to be in the right place at the right time. In order to do this, it is necessary to pick up and use perceptual information that specifies the time to arrival of an object at an interception point. In the present study, we examined the ability to intercept a laterally moving virtual sound object by controlling the displacement of a sliding handle and tested whether and how the interaural time difference (ITD) could be the main source of perceptual information for successfully intercepting the virtual object. The results revealed that in order to accomplish the task, one might need to vary the duration of the movement, control the hand velocity and time to reach the peak velocity (speed coupling), while the adjustment of movement initiation did not facilitate performance. Furthermore, the overall performance was more successful when subjects employed a time-to-contact (tau) coupling strategy. This result shows that prospective information is available in sound for guiding goal-directed actions.

## Introduction

Successfully intercepting a moving object depends on a strong spatiotemporal connection between the individual's movement and the movement of the object to be intercepted. For example, a baseball outfielder must pick up and use visual information that specifies the future arrival position of the ball to allow them to get to the right place at the right time to successfully complete the catch (McBeath et al., 1995). Through the principles of perception-action coupling, tuning into the relevant sensory information that specifies the properties of the movement of an object through the environment should ensure that the regulations of subsequent movements are prospectively controlled (Lee et al., 2001). While vision has been the sense that is predominantly studied in object interception (Port et al., 1997; Lee et al., 2001), it may also be possible to intercept an object by using the sound it makes when moving. In principle, auditory interception explains how visually-impaired “Goalball” players locate a sounding soccer-sized ball as it moves through space, yet little is known about the strategies that individuals use to guide their actions when relying on auditory information alone.

When intercepting an object, one of the main variables that can be used to guide movement is time-to-arrival (tau) (Lee, 1998). In summary, tau is the time-to-closure of a gap (e.g., angle, velocity, pitch, force) at its current closure rate. This invariant has been hypothesized to be sufficient to guide actions prospectively, irrespective of the origins of the sensory information (e.g., light, sound, heat etc.) (Lee, 1998). In terms of using sounds, some studies have suggested that the acoustic time-to-contact might also be used by humans to detect the arrival of objects (Rosenblum et al., 1987, 2000; Guski, 1992; Ashmead et al., 1995). Although Shaw et al. (1991) found that increasing loudness is a useful acoustic parameter that specifies the time to arrival of a constant-intensity sound as it moves toward a listener, Guski (1992) suggested that one should also consider other information sources such as the rate-of-change of sound intensity and the distance of the sound source, which may be specified by the spectral content of a sound.

In humans, the anatomical positioning and structure of the auditory receptors contribute to the relative spatial location of sounding objects. The three primary cues that are thought to be used for auditory localization are: (i) interaural time difference (ITD), which is the difference in arrival time of a sound wave to each ear; (ii) interaural level difference (ILD), which is the difference in the pressure of sound arriving at each ear; and (iii) head-related transfer function (HRTF), which is the unique way a sound spectrum is filtered by the physical properties of an individual's pinna, head and torso (Hebrank and Wright, 1974; Shaw, 1974). The first two (ITD and ILD) are binaural and are based on the mismatch that the sound signals have when reaching the left and right ears. Sounds that are off to one side reach one ear before the other and are louder at one ear than the other due to auditory occlusion caused by the head. Behavioral research, in which listeners judge sound locations as ITD varies, have indicated that this variable is used as an effective cue for defining the location of low-frequency sounds (Wightman and Kistler, 1997, 1998). ILD, on the other hand, provides useful binaural information about the location of sound sources with a high frequency but not a low frequency (Shaw, 1974). The combination of ITD and ILD, while very efficient in allowing the sound location along the azimuth coordinates (positions left to right), are more ambiguous in detecting the elevation of a sound source (positions up and down). By incorporating the HRTF, localization limitations inherent when using ITD and ILD cues alone can be overcome (Hofman et al., 1998). Empirically it has been shown that some mammals (including humans) appear to use all three auditory cues (ITD, ILD and HRTF) to localize sounds (for a review, see Grothe et al., 2010).

In order to guide our movement to intercept a sound source, we need to have a reliable source of information that tells us the direction in which the sound is traveling and yet can guide the online control of interception action. It has been suggested that the increase in the binaural rate of intensity (or loudness growth) of a sound is the primary information source used to determine the trajectory and velocity of an approaching sound emitting source (Shaw et al., 1991). Although ILD seems to be an advantageous auditory cue for the localization of an approaching sound, the sound localization in the horizontal plane (azimuth) involves both ITD and ILD binaural cues. Research has shown that the ITD can be used by the mammalian auditory system to compute the angle of incidence of an acoustic sound source with low frequencies (less than 2 kHz) on the horizontal plane, while the ILD was found to be not as informative when the frequency of the sound source is low (Yin, 2002). Therefore, in a situation where a sound-source with low frequency is rotating around the listener, the ITD is a more plausible candidate auditory variable that could be used by the perceiver to regulate interceptive actions. In this study, we aimed to test what strategies individuals could inclusively adopt in order to intercept a low-frequency rotating sound. One might need to adjust the initiation of the movement, vary the duration of the movement or alter the speed of movement to accomplish the task. Moreover, we wanted to investigate whether prospective temporal information (tau-τ) is used to correctly guide the interceptive movement.

Tau is the time to closure of any action gap (τ_*X*(*t*)_) and is defined as the ratio between the magnitude of the gap and its current rate of change: *X*/*Ẋ*. In general, it is theorized that the way the variable tau is used to guide movements is through the principle of τ-coupling which considers the closing of two physical gaps synchronously (*X* and *Y*) by keeping the τs of the gaps in constant ratio *k*, so that τ_*x*_ = kτ_*y*_. For instance, Lee et al. (2001) showed that when subjects moved to temporally intercept a moving object in a pre-designated target zone, they τ-coupled the closure of the gap between the hand and the moving object with the closure of the gap between the hand and the target zone where temporal interception was to take place. In this study, we used a similar set-up but used a moving sound instead of a moving visual object. In this case, the defined action gaps are: (1) the gap between the initial position of the sound and the intercepting point (target zone) that is specified by changes in the ITD (τ_*ITD*_) and (2) the gap between the starting hand position and the target zone (interception point) (τ_*m*_). As in the Lee et al. study (2001), it is possible to successfully perform the task by keeping these two taus coupled at a constant ratio (τ_*m*_ = *k*τ_*ITD*_) in a way that both gaps will reach 0 (close) at the same time. An alternative strategy to temporally intercept moving sound objects could be to alter the initiation time depending on the speed of the sound and keep the duration/velocity of the movement constant across different sound speed profiles. If the movement profile is altered depending on the speed of the sound, this will be reflected in the duration and/or the velocity of intercepting movements. It has been shown that intercepting visual moving targets required people to hit fast-moving visual targets more quickly than slow ones, which suggests the coupling between the speed of movement and target (e.g., Van Donkelaar et al., 1992; Brenner et al., 1998; Brouwer et al., 2005). To test which strategies are really applied, we simulated 5 different sound sources each having different speeds moving along the interaural axis (i.e., inside the head), from the left to the right side and from the right to the left side of the participants. The participants were instructed to intercept the virtual sound source in a target zone that was located directly in front of them. We examined some of the strategies that the participants adopted to temporally intercept the moving sound objects.

## Methods

### Ethics statement

The experimental protocol was approved by the members of the Ethics Committee of School of Psychology, Queen's University Belfast. Prior to entering the study, all the participants provided their written informed consent, which had been approved by the institutional review board.

### Participants

Ten healthy, right-handed adults (five females; aged 26.1 ± 5.7 years) with normal hearing and no history of neurological disorders participated in the experiment.

### Materials and apparatus

**Figure 1:**
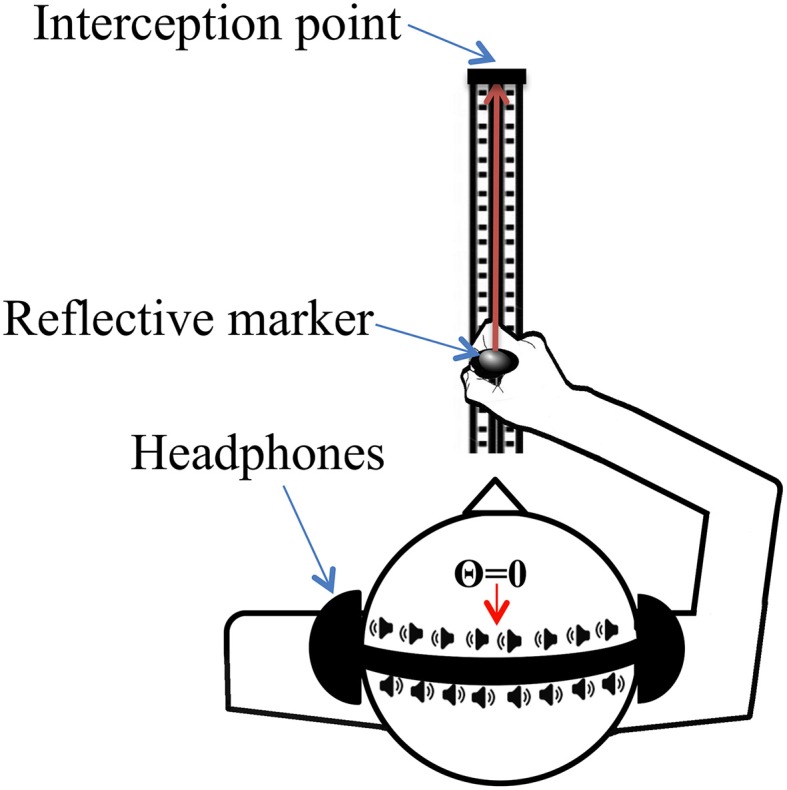
**Experimental setup**. Participants moved the handle toward the interception point to temporally intercept the sound moving along the interaural axis (i.e., inside the head). In order to simulate the motion of the sound, timing presentation of sounds to either ear in the headphones was adjusted to create varying ITD information. The movement of the sound is represented by the speaker icons in the diagram.

Each participant sat with their eyes closed and held in his/her hand a handle that was mounted on a straight 50 cm railing. The handle was positioned in front of them and could be slid forwards and backwards along the rail. The start and interception points were fixed so that the distance traveled remained constant across all trials and participants. To measure the kinematics of the hand movement, a reflective passive marker was placed on the top of the handle (See Figure 1). The movement of the reflective marker was recorded at 500 Hz by three Qualisys Oqus 300 Motion Capture cameras connected to a Dell PC running Qualisys Track Manager (QTM) software (www.qualisys.com). The spatial accuracy was estimated to be close to ±0.1 mm. Each sound was programmed to start to play 1 s after participants moved the handle from a rest position to its starting position.

The sound stimuli were generated using a Pure Data (http://puredata.info/) patch, and delivered through noise-isolating headphones at a constant volume level (68 dB SPL). The participants were instructed to slide the handle along the rail so as to intercept the sound at the end. It should be noted that because of the nature of the stimuli, the hand movement and the sound trajectory were never intersected, which means that the interception was “virtual” and “temporal” rather than “positional.” Successful temporal interceptions were reinforced through additional auditory feedback consisting of a short (400 ms) tone dropping in frequency from 1600 to 200 Hz and fading out. For a trial to be considered successful, the handle had to arrive in the target zone within a time window which was calculated for each sound differently as ±9° spatial deviation from the center of the interception zone; trials outside this window were considered as unsuccessful. A trigger to send a single-pulse signal was installed in the target end of the rail to detect the arrival of the handle.

Given the abstract nature of the task, it was important that participants were given some training. During the training phase, the time window was increased by steps of 50 ms until they were able to temporally intercept the sound twice in a row. After two consecutive successful trials, the time windows were then decreased by steps of 50 ms until the movement was successfully performed within the experimental time window. Each participant was required to perform 10 successful trials in a row in order to move to the next sound that was randomly assigned. All the participants completed this training phase in less than 5 min.

### Stimuli

A total of 5 rotating auditory stimuli were simulated [accelerating (ACC); decelerating (DEC); constant velocity fast (Vf); constant velocity medium (Vm); constant velocity slow (Vs)]. The sounds were created using MATLAB (The Mathworks Inc., 2011) by varying the ITD as computed using equation (1) adopted from (Kuhn, 1977). Sound stimuli consisted of pure tones with 200 Hz sound pulses of 45 ms duration each. In order to create fast, medium and slow sound velocities, the stimuli were composed of bursts of pulses rotating from θ = −*pi*/2 to θ = *pi*/2 with different inter-pulse intervals (0.0227, 0.1134, and 0.1587 s) with 1.42, 3.33, and 4.28 s durations and with 126.76, 54.04, 42.05 degrees/s of angular velocity, respectively. The stimuli were saved as WAV files at 44.1 kHz and 16 bits.

Inter-aural time differences were calculated according to the following equation:

(1) $$ITD\left(t\right)=3\;\frac{r}{v}\;sin\theta_{i}$$

where: *r* (8.75 cm) is the approximate size radius of the typical human head (Algazi et al., 2001), *v* is the speed of sound in room-temperature air (343 m/s) and θ_*i*_ is the sound source's deviation angle (from −π/2 to π/2 with the step of π/20).

For constant accelerating/decelerating stimuli, the sound was accelerating/decelerating from θ = −π/2 to θ = 0 and then decelerating/accelerating to θ = π/2. In order to simulate the accelerating-decelerating sounds, the inter-pulse intervals were decreasing from 0.045 s (θ = −π/2) to 0.0045 s (θ = 0) and then increasing to 0.045 s (θ = π/2) linearly with a step of 0.0045 s. The ordering of decreasing and increasing inter-pulse intervals were reversed for the decelerating-accelerating sounds. The duration of both of the sound trajectories were 2 s.

### Data processing

The raw positional data were filtered using a second order low-pass Butterworth filter with a cut-off frequency of 8 Hz using MATLAB (The Mathworks Inc. 2011). The displacement vector computed from when the handle clearly started to move was defined as the moment when the handle velocity reached 10% of its peak velocity on that trial to the moment when the handle velocity dropped to 10% of its peak velocity just before reaching the end of the trial. For the analysis, the main variables considered included the followings: success rate, the strength of the coupling between the information (ITD) and the movement (handle to end point) taus, movement initiation, movement duration, peak velocity and time to peak velocity.

#### Success and fail rate

The percentages of trials where subjects were successful or unsuccessful when intercepting the moving virtual target were calculated for each sound condition separately.

#### Test of prospective (τ-coupling) strategy

**Figure 2:**
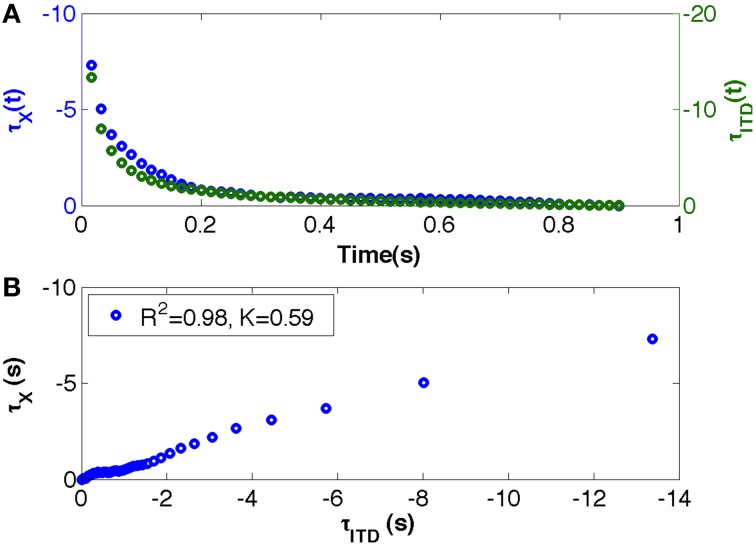
**(A,B)**. An example of an almost perfect tau coupling between the tau of the movement gap and the tau of the ITD during interceptive movement collected from a participant in the Vm condition. **(A)** The blue dots represent the tau of a motion-gap τ_*M*_ (participant's movement) that is hypothesized to be coupled with informational tau τ_*ITD*_ represented with green dots; τ_*M*_ and τ_*ITD*_ are plotted against movement time vector. **(B)** τ_*M*_ is plotted against τ_*ITD*_ and *r*^2^ of the τ-coupling linear regressions (*r*^2^ = 1 if coupling perfect), *K* is the constant value in the coupling equation τ_*M*_ = *k*τ_*ITD*_.

We tested whether the interceptive movement was accomplished by keeping the tau of the movement coupled to the tau of ITD at a constant ratio (τ_*M*_ = *k*τ_*ITD*_). In order to have the same number of data points for the two time series (τ_*M*_ and τ_*ITD*_), the θ_*i*_ was time-binned from −π/2 or π/2 to 0 using bin durations of π/2*T* − 1 in which *T* is the length of the movement time vector. For each sound, the tau of the ITD was calculated as the time-to-closure of the ITD gap at its current closure rate. To express it symbolically, suppose that at time *t*, the size of an ITD is *ITD*(*t*) and its rate of change is *IṪD*(*t*). Then tau of the ITD-gap at time *t* is written as τ_(*ITD,t*)_ and this equals *ITD*(*t*)/*IṪD*(*t*) as calculated in Equation (1). The *ITD*(*t*) vectors for individual vectors were calculated using Equation (1). For constant velocities, the *IṪD*(*t*) was computed by calculating differences between adjacent elements of *ITD*(*t*) and dividing it by *dt*, which was 1/sampling frequency (1/500). For the constant acceleration and deceleration, *IṪD*(*t*) was obtained by calculating differences between adjacent elements of *ITD*(*t*) and dividing it by the time vector *dt* which was derived from the linear interpolation of the displacement time vector and the vector of the inter-pulse intervals that were used to create the two sounds. Accordingly, tau of a motion-gap (τ_*M*_) was computed by dividing the current size of the motion-gap, *x*, by its current rate of closure to give the first-order time to closure of the motion-gap. Finally, the strength of the τ_*M*_ = *k*τ_*ITD*_ (τ-coupling) was measured on each trial by the *r*^2^-values of the linear regressions through the “straight” sections of the τ_*M*_ vs. τ_*ITD*_ plots. The strength of tau coupling was calculated for all the trials (successful and unsuccessful) (See for example Figure 2). To see the grand average tau profiles, we first normalized the tau profiles (τ_*M*_ and τ_*ITD*_) between 0 and 1 and then interpolated to give 101 data points. After that, the averaged normalized tau of movement across all the participants was plotted against averaged normalized tau of ITD (See **Figure 4**).

#### Movement initiation

The moment of the initiation of the interceptive movements was computed as the time that elapsed between when the sound started to play and the beginning of the movement, defined as when the handle velocity reached 10% of peak velocity for that particular trial.

#### Movement duration

The movement duration was calculated as the time that elapsed when the handle velocity exceeded 10% of peak velocity, and when it fell back below 10% of peak velocity.

#### Peak velocity

Peak velocity was calculated as the maximum value of the absolute velocity of each interceptive movement.

#### Time to reach peak velocity

The time to reach peak velocity for each intercepting movement was computed as the time that elapsed from the start of the handle movement to reach its peak velocity.

To visualize the grand average velocity profiles, we normalized the velocity profiles between 0 and 1 and then interpolated to give 101 data points. After that, the averaged normalized velocity profiles across all the participants was plotted against the normalized time (See **Figure 8**).

## Statistical analysis

The experiment contained two factors: Sound types (accelerating, decelerating, fast, medium, and slow, or ACC, DEC, Vf, Vm, and Vs, respectively) and Successful/Unsuccessful (S/U) trials. We used One-Way repeated measures ANOVA to analyse the results of success rates and a Two-Way ANOVA with repeated measures to analyse the results of the rest of the variables. *Post-hoc* comparisons were performed by means of *t*-tests applying the Bonferroni correction for multiple comparisons when required. A partial-eta-squared statistic served as the effect size estimate.

### Results

#### Success rates

The average success rate was 35.8 ± 10.1 for the accelerating, 47.4 ± 15.1 for the decelerating, 49.6 ± 17.8 for fast, 48.6 ± 16.4 for the medium and 40.7 ± 16.1 for the slow sound. There was no statistically significant difference between the percentage of successful trials among different sound types as determined by a one-way ANOVA [*F*_(4, 45)_ = 1.499, *p* = 0.218, η^2^ = 0.12].

#### Strength of τ-coupling

To test whether sound interception might be achieved by following an information-movement coupling strategy, we examined the extent of the coupling between the hand movement and the auditory information. We measured the strength of coupling (r-squared values of linear regressions) when the tau of the movement was plotted against the tau of ITD (See for example Figure 2B).

**Figure 3:**
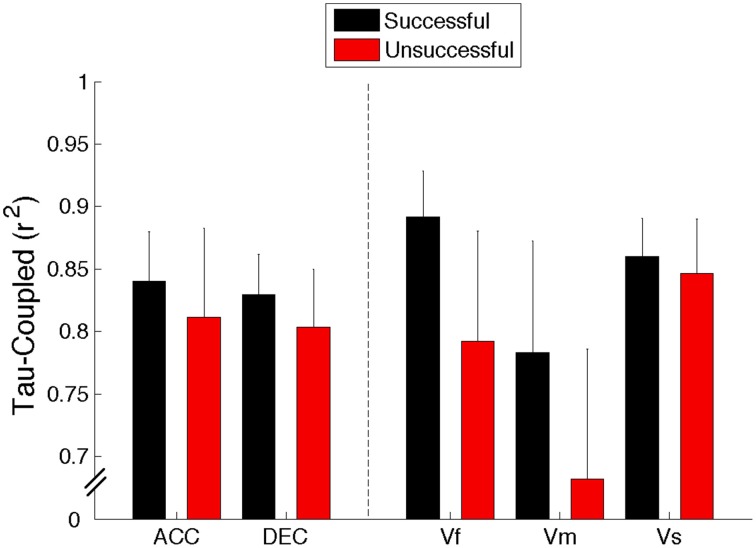
***R*^2^ of tau-coupling regression for successful and unsuccessful trials averaged across all 10 participants for all the sounds (ACC, Accelerating; DEC, Decelerating; Vf, Fast; Vm, Medium; and Vs, Slow)**. Error bars denote standard errors.

A Two-Way, repeated-measures ANOVA (5 Sounds × 2 S/U) revealed that overall the *r*^2^-values were significantly greater for the successful trials as compared to the unsuccessful ones [*F*_(1, 9)_ = 6.007, *p* = 0.037, η^2^ = 0.4]. The results showed neither a significant main effect of sound trajectory [*F*_(4, 36)_ = 0.727, *p* = 0.579, η^2^ = 0.07] nor a significant interaction between sounds and the S/U condition [*F*_(4, 36)_ = 0.529, *p* = 0.715, η^2^ = 0.05]. This result supports the hypothesis that the movement of the effector (handle) was guided mainly using a τ-coupling strategy as τ_*M*_ and τ_*ITD*_ were kept in a constant ratio in order to accomplish the task (Results are summarized in Figure 3).

**Figure 4:**
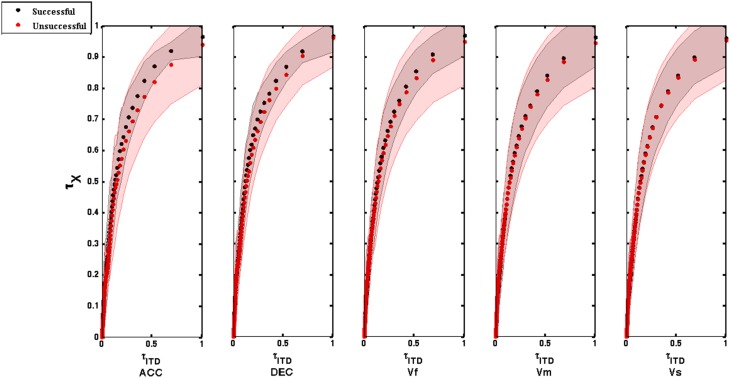
**Tau coupling profiles for successful and unsuccessful trials across 10 participants for all the sounds (ACC, Accelerating; DEC, Decelerating; Vf, Fast; Vm, Medium; and Vs, Slow)**. The averaged normalized tau of movement is plotted against averaged normalized tau of ITD. The tau profiles were normalized between 0 and 1 and then interpolated to give 101 data points. Shaded regions around the tau coupling profiles represent error bars (SEM).

#### Movement initiation

In order to determine whether the initiation of the movement varied across conditions and whether this may be the main factor influencing the participants' performance, movement initiation times were computed for each trial as the time that elapsed between when the sound started to play and the beginning of the movement.

A Two-Way repeated measures ANOVA (5 Sounds × 2 S/U) revealed no significant main effect for Sound types [*F*_(4, 36)_ = 2.866, *p* = 0.109, η^2^ = 0.24], the S/U condition [*F*_(1, 9)_ = 0.995, *p* = 0.345, η^2^ = 0.1] or interaction [*F*_(4, 36)_ = 2.107, *p* = 0.1, η^2^ = 0.19]. This implies that participants did not use the initiation of interceptive movements as a main strategy to accomplish the task.

#### Movement duration

Movement duration was computed for each interceptive movement to see whether it stayed constant or varied across conditions and is defined as the time that elapsed from the start of the handle movement to the end of the interceptive movement.

A Two-Way repeated measures ANOVA (5 Sounds × 2 S/U) revealed a significant main effect for Sound types [*F*_(4, 36)_ = 15.666, *p* < 0.001, η^2^ = 0.63]. Simple main effects analysis showed that movements had significantly shorter durations when intercepting the accelerating sounds compared to sounds with medium (*p* < 0.001) and slow velocities (*p* = 0.016). Moreover, the movement time was significantly shorter for fast speed sounds compared to slow (Vs) and medium (Vm) speed sounds (*p* = 0.009 and 0.006, respectively). This shows that the movement duration varied as a function of the sound's velocity and that the patterning of information influenced subsequent movements.

**Figure 5:**
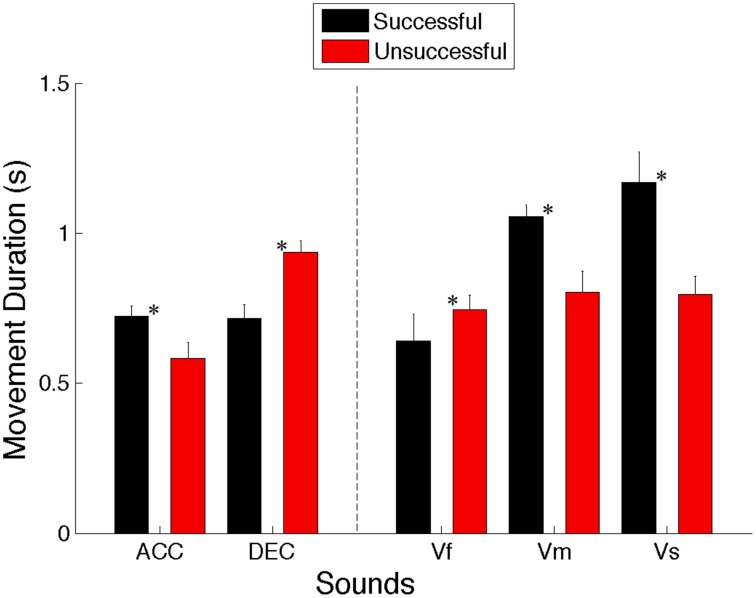
**Movement times for successful and unsuccessful trials averaged across all 10 participants for all the sounds (ACC, Accelerating; DEC, Decelerating; Vf, Fast; Vm, Medium; and Vs, Slow)**. Error bars denote standard errors. Significant comparisons between conditions are highlighted with an asterisk (^*^p < 0.05).

A significant main effect for the successful/unsuccessful factor [*F*_(1, 9)_ = 11.878, *p* = 0.007, η^2^ = 0.57] was also found, with overall faster movements being found for the successful trials compared to the unsuccessful ones. However, it should be noted that this trend was not the same for all the sounds (See for example Figure 5).

There was also a significant interaction between the sound and successful/unsuccessful factors [*F*_(4, 36)_ = 33.609, *p* < 0.001, η^2^ = 0.79]. *Post-hoc* analysis indicated that for successful trials the movement time was shorter for accelerating sounds compared to fast (Vf) (*p* = 0.014), medium (Vm) (*p* < 0.001), and slow (Vs) (*p* = 0.01) sounds, and also for decelerating sounds compared to medium (*p* < 0.001) and slow (*p* = 0.005) sounds and for the fastest sound compared to medium (*p* < 0.001) and slow (*p* = 0.001) sounds. Moreover, for unsuccessful trials, the movement time was shorter for accelerating compared to decelerating (*p* = 0.004) and fast (*p* = 0.023) sounds. Importantly, our results revealed that moving for a longer duration to intercept the accelerating, medium and slow sounds led to higher rate of successful trials compared to unsuccessful ones (*p* = 0.002, 0.001, and 0.001, respectively), while successful trials for decelerating and fast sounds were achieved by shorter movement durations compared to the unsuccessful ones (*p* < 0.001).

Overall, these results indicate that in spite of the same amplitude of movements to be made, movement durations are not kept constant across conditions. This suggests that the kinematics adopted are being influenced by the dynamics of the moving sound object so as to successfully intercept it in the target zone (cf. Figure 5).

#### Peak velocity

To assess whether altering the hand velocity benefits participants' performance or not, peak velocity was calculated from each individual intercepting movement.

**Figure 6:**
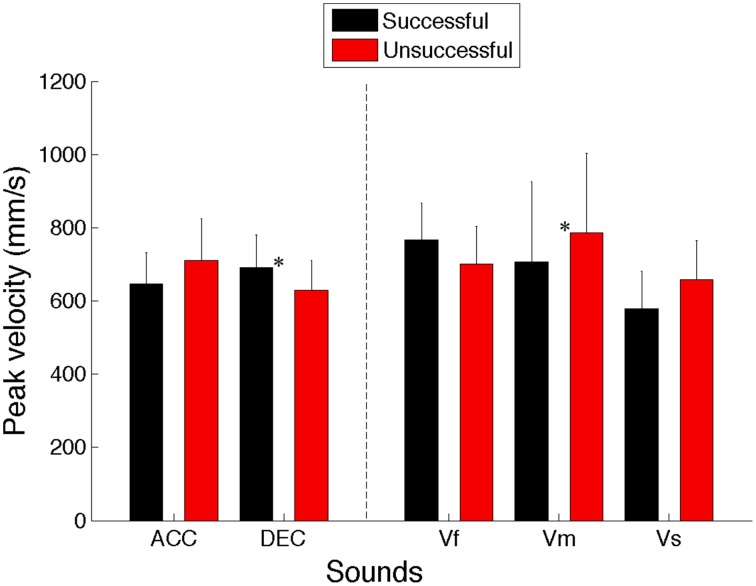
**Averaged peak velocities for successful and unsuccessful trials across all 10 participants for all the sounds (ACC, Accelerating; DEC, Decelerating; Vf, Fast; Vm, Medium; and Vs, Slow)**. Error bars denote standard errors. Significant comparisons between conditions are highlighted with an asterisk (^*^p < 0.05).

A Two-Way repeated measures ANOVA (5 Sounds × 2 S/U) revealed no significant main effect for Sound types [*F*_(4, 36)_ = 0.342, *p* = 0.847, η^2^ = 0.037] or the S/U condition [*F*_(1, 9)_ = 0.928, *p* = 0.361, η^2^ = 0.093]. There was a significant interaction between the sound and successful/unsuccessful factors [*F*_(4, 36)_ = 5.5, *p* = 0.001, η^2^ = 0.379]. Importantly, post-hoc analysis revealed that successful trials for decelerating sounds were achieved by larger peak velocity compared to the unsuccessful ones (*p* = 0.006), while moving with lower peak velocity to intercept the medium sound led to higher rate of successful trials compared to unsuccessful ones (*p* < 0.01). This implies that the change in the peak velocity was selectively advantageous for participants to accomplish the task (cf. Figure 6).

#### Time to peak velocity

A Two-Way repeated measures ANOVA (5 Sounds × 2 S/U) revealed a significant main effect for Sounds types [*F*_(4, 36)_ = 15.872, *p* < 0.001, η^2^ = 0.638]. *Post-hoc* analysis showed that movements had significantly shorter time to reach the peak velocity when intercepting the accelerating compared to decelerating (*p* < 0.005), medium (*p* < 0.005) and slow sounds (*p* = 0.017). Moreover, the time to reach the peak velocity was significantly shorter for fast compared to decelerating (DEC), slow (Vs) and medium (Vm) speed sounds (*p* = 0.017, 0.018, and 0.03, respectively). This implies that the time to reach the hand peak velocity changed as a function of the sound's velocity. Importantly, no significant effect was found for the successful/unsuccessful factor [*F*_(1, 9)_ = 0.565, *p* = 0.471, η^2^ = 0.059].

Moreover, there was a significant interaction between the sound and successful/unsuccessful factors [*F*_(4, 36)_ = 16.790, *p* < 0.001, η^2^ = 0.651]. *Post-hoc* analysis indicated that successful interception of accelerating and fast sounds needed shorter to reach peak velocity than the unsuccessful ones (*p* < 0.001 and < 0.01, respectively), while successful interception of decelerating and medium sounds required longer time to peak velocity than unsuccessful attempts (*p* < 0.005). Moreover, for successful trials the time to reach the peak was shorter for accelerating sounds compared to decelerating (DEC) (*p* < 0.001) medium (Vm) (*p* < 0.005) and slow (Vs) (*p* < 0.01) sounds, and for the fastest sound compared to decelerating, medium (*p* < 0.05) and slow sounds (*p* < 0.01). No significant difference was found for unsuccessful trials across different sounds.

**Figure 7:**
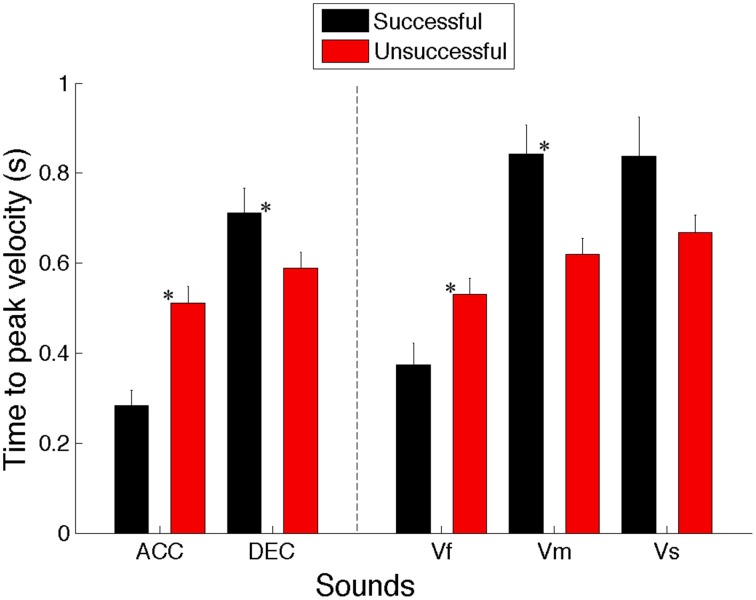
**Averaged time to peak velocities for successful and unsuccessful trials across all 10 participants for all the sounds (ACC, Accelerating; DEC, Decelerating; Vf, Fast; Vm, Medium; and Vs, Slow)**. Error bars denote standard errors. Significant comparisons between conditions are highlighted with an asterisk (^*^p < 0.05).

Overall, these results show that although movements' amplitude was the same, the time to reach the hand peak velocities is not kept constant across different conditions, which implies that the dynamics of the moving sound object influenced the velocity profile adopted to successfully intercept it (cf. Figure 7).

## Discussion

The aim of this study was to investigate if and how participants can intercept a moving virtual sound object in a pre-designated target zone. By varying the dynamics of the information (the moving sounding object), we wanted to see the resulting effects on the movement behavior. The sounds used were found to be of a similar level of task difficulty (as demonstrated by the similar success rates obtained by the participants), but by presenting different physical characteristics (accelerating, decelerating, and being fast, medium and slow in velocity) they were found to significantly influence the form of the interceptive movement made by the participants. Here we showed that the dynamics of the sound significantly influenced movement duration and form across the different experimental sounds.

Following tau theory (information-movement coupling), we assumed that the control of movement would involve closing two gaps in synchrony: an information gap (defined by the ITD gap to the interception point) and a movement gap (defined by the gap between the hand and the target zone). Although the average strength of tau coupling for each sound condition was higher for successful rather than unsuccessful trials (for example, for the fastest sound the mean *r*^2^-value is 0.9 for successful and 0.8 for the unsuccessful trials with *p* = 0.07) these differences were not found to be significantly different (See Figure 3). Moreover, in Figure 4 it can be seen that the averaged normalized tau-coupling profiles for the successful and unsuccessful trials had similar shapes, but the successful ones have slightly larger slopes, and that the profile of couplings seem to be less variable as compared to unsuccessful ones. This suggests that the participants adjusted temporal pattern of movements in order to successfully intercept the sounds. Overall, it appears that coupling the tau of the movement onto the tau of the ITD was a successful strategy applied to accomplish the task.

Furthermore, the changes in movement initiation times were not used preferentially for achieving a better performance. In contrast, analysis of movement duration yielded different results; for each sound condition there was a significant difference between successful and unsuccessful trials (See Figure 5). For decelerating and fast sounds, the movement duration tended to be shorter for successful trials, whereas for accelerating and slow and medium speed of sounds the movement duration tended to be shorter for unsuccessful trials. Thus when the virtual target travels initially at high velocity and then decelerates, and when it travels at high constant velocity, the unsuccessful attempts happened mostly when subjects intercepted the target too late, so the handle movement duration was too long. However, when the target traveled initially at a low velocity and then accelerated, and when it traveled at slow and medium constant velocities, the participants missed the virtual target because the handle arrived too early at the interception point; in other words, handle movement durations were too short. Moreover, from Figure 5 it can be seen that for sounds with constant velocity, ballistic (constant duration) movements were less successful than sound-adapted movements. Thus, our results highlight the need to adjust movement duration to the information specifying the moving auditory object's time to arrival in the target zone in order to successfully intercept it.

**Figure 8:**
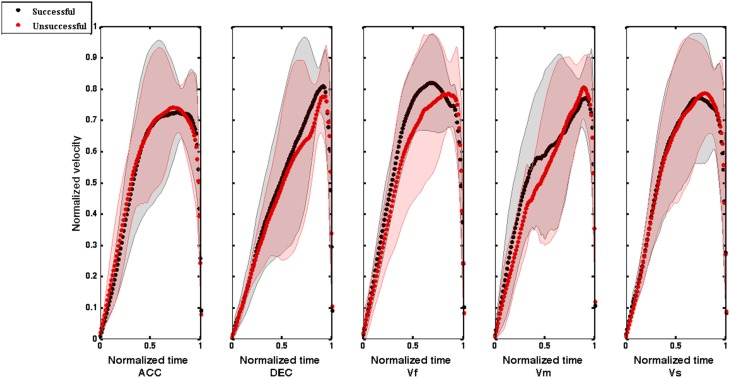
**Averaged normalized velocity profile plotted against normalized time for successful and unsuccessful trials across all 10 participants for all the sounds (ACC, Accelerating; DEC, Decelerating; Vf, Fast; Vm, Medium; and Vs, Slow)**. The velocity profiles were normalized between 0 and 1 and then interpolated to give 101 data points. Shaded regions around the velocity profiles represent error bars (SEM).

The analysis of peak velocity and time to peak velocity gave us a better picture of the dynamic of hand velocity during interceptive movements. It turns out that successful trials for decelerating sounds were achieved by increasing the maximum velocity compared to the unsuccessful ones, while the reverse was true for the medium speed sounds. Moreover, it appears that the time to reach the peak velocity was significantly shorter for fast and accelerating sounds compared to decelerating, slow and medium speed sounds. This reflects that participants coupled the speed of their movement to the speed of the virtual target to successfully intercept it. Additionally, in the Figure 8 it can be seen that the standard errors around the velocity profiles are large for both successful and unsuccessful trials, indicating variability in the shape of velocity profile. However, it seems that averaged normalized velocity profiles for successful and unsuccessful trials are different in form. For instance, while movements with more circular velocity profiles are beneficial for intercepting accelerating, fast and slow speed sounds, interception of decelerating and medium speed sounds resulted in movement with increases in velocity toward the very end of the movement. This implies that intercepting sounds with different velocities require using different strategies for selecting the profile of movement velocity. In other words, the velocity of interceptive movements is adjusted based on the speed of the moving sound sources. Overall, we showed that adjusting the movement velocity parameters such as the maximum speed (peak velocity) and time to peak velocity assisted participants to successfully intercept the sounds. This suggests that dynamic information specifying the speed of the sound provided by changes in ITD entail the adjustment of different hand velocity profiles. This is in accord with other studies about intercepting moving targets which show that people hit fast targets more quickly than slow ones, which suggests coupling between the speed of movement and target (e.g., Van Donkelaar et al., 1992; Brenner et al., 1998; Brouwer et al., 2005).

It might be the case that each speed condition leads to a prediction of the time-of-arrival of the virtual object at the interception location, which would be met with different arm movement durations and speeds. Higher constant speeds lead to faster interception movements, and for the accelerating/decelerating conditions (both of which take the same time to reach the interception location) it leads to equal movement durations. Interestingly, Port et al. (1997) also found that when intercepting visual targets moving with different velocities, the constant timing errors tended to be negative (early interception) for decelerating targets, vs. positive constant timing errors (late interception) for accelerating and constant velocity targets. They inferred that for the decelerating conditions subjects were not able to compensate for the speed decrement and intercepted the target too early, while for accelerating conditions participants were unable to compensate for the speed increment which resulted in late interceptions (Port et al., 1997). Overall, our results revealed that movement durations and velocity variables such as peak velocity and time elapsed before reaching the peak velocity varied significantly in spite of the distance to cover remained constant. These variations in movement duration and form were in spite of the planning and initiation parts of the movement being comparable across conditions. This suggests that to accomplish the task, subjects tended to use a prospective (τ-coupling) and speed-coupling strategy where the information specifying the time to arrival (tau) and the speed of the sounding object guided the interceptive action.

Although it has already been shown that visual tau (Lee et al., 2001) and speed coupling strategy (e.g., Van Donkelaar et al., 1992; Brenner et al., 1998; Brouwer et al., 2005) can be used to control interceptive movements, here we showed that interceptive action could be guided using an acoustic variant, i.e., the specification of lateral motion along the interaural axis using the tau of the ITD. Previous research has shown that at least three types of acoustic variables can allow listeners to reliably indicate when a moving sound source is passing: the ITDs, the Doppler effect and the intensity changes (Rosenblum et al., 1987), with individuals being highly accurate when all three acoustic cues are available. Although in the real world all the acoustic cues are readily available, here we have shown that by just considering the rate of changes of one of them (the ITD), individuals were able to control their movements and intercept a moving sound source quite successfully. The stimuli applied in the study were low in frequency (200 Hz) with the ITD being shown to be indeed an effective way of defining the objects' location at this frequency of sound (Wightman and Kistler, 1997, 1998). In contrast, neither the ILD nor HRTF location cues were provided; hence the sound was perceived as moving only along the interaural axis, i.e., inside the head. Moreover, in this experiment the change in stimuli velocity was simulated by changing the pulse rate; hence, the participants could have adjusted their movements using pulse rate rather than a spatial cue. Interacting with a high frequency sound source moving in a three-dimensional space would involve ILD and the HRTF as well. Nonetheless, the same methodological approach employed here can be used to evaluate other acoustic variables combined or in isolation could, in principle, be a reliable means for guiding movements so as to interact with a traveling sound source.

It is theoretically possible that participants relied on different strategies to successfully intercept a moving sound source. However, since in this experiment the initial position of the effector (handle) and the point of interception was the same for all the participants and across all trials, the main variables involved in this task were related to movement time and form such as movement initiation, duration, tau coupling, peak velocity and time to peak velocity. Our results showed that while people could have used different strategies to intercept the moving sound, the overall performance was more successful when they employed a tau coupling strategy and coupled the velocity of the movement differently to the velocity of the virtual target. This implies that using a prospective temporal and velocity information strategy provided by the rate of change of the ITD is more efficient than using the current position of the sound to compute when to initiate the interceptive movement. Interestingly Port et al. (1997) found that when intercepting visual moving targets, participants accomplished the task by using different strategies. They proposed that the selection of a particular strategy depends on factors included within the experimental paradigm such as the target velocity or even individual differences. Addressing the same question, Van Donkelaar et al. (1992) asked participants to intercept moving visual targets as fast as possible and found that in order to accomplish the task, participants initiated their movement earlier as the target velocity increased. It should be noted, however, that in these studies the emphasis of the task was on the speed of the response rather than on its accuracy, and consequently the participants were highly constrained in the strategy they could adopt. Similar to the study conducted by Port et al. (1997), in the present study there were no explicit instructions given regarding when to initiate the movement, which would allow the participants to select the strategy they deemed most appropriate.

Overall, our results show that reliance on a perception-action coupling strategy (tau-coupling) was preferred in this temporal interception task over a variable movement initiation strategy and constant movement duration. Indeed, our results showed that for the constant velocity conditions, participants produced similar average movement durations in unsuccessful trials, but not in successful ones (Figure 5). These findings are in accordance with the results obtained from experiments on infants (van der Meer et al., 1994; van Hof et al., 2006) and children with Autism (Whyatt and Craig, 2013) in which catching/intercepting moving objects was found to be more successful when interceptive movement was coupled online to the sensory information that directly specifies the time to ball arrival. Other research has shown that a tau-coupling strategy is used in a diverse range of goal-directed actions (Craig and Lee, 1999; Lee et al., 1999, 2001; Craig et al., 2000a,b, 2005). Moreover, our results revealed that the movement durations and velocity variables such as peak velocity and time to reach the peak velocity varied significantly in spite of the distance to cover remained constant. It has been suggested that the difference between velocity profiles during intercepting movements was resulted from coupling the velocity of the hand to the velocity of the target (Brenner et al., 1998; Brouwer et al., 2005). Overall, our results imply that both tau and speed coupling increase the precision of intercepting movement.

The results of the current study contribute to the aforementioned line of research by showing that tau and speed coupling can be used to intercept a moving sound source even when the information available is just the time differences of sound arriving at each ear (ITD). Interestingly, this study shows how a simple variable such as the ITD can provide sufficient information about the lateral time to arrival of a sounding object. By sensing and using the tau of the ITD (the time to-closure of the ITD gap at its current closure rate) and changes in its velocity (speed coupling), the participants could successfully guide the closure of a movement gap to intercept the sound in a pre-designated target zone.

While the results of this study are promising, potential limitations should be noted. First, the sound stimuli used in the current experiment were created on the basis of an isolated binaural cue (i.e., dynamic changes in ITD) and were presented to the participants via headphones, which resulted in the perception of the sound stimuli moving along the interaural axis (i.e., inside the head). This led us to investigate how a simple variable such as the ITD can provide sufficient information about the lateral time to arrival of a sounding object for successfully intercepting it. By doing this, we avoided the presence of potentially conflicting spatial information associated with free-field sound sources, which in turn resulted in lowering the ecological validity of our experiment. For instance, during the experiment the hand movement and the sound trajectory were never intersected, which means that the interception was “virtual” and “temporal” rather than “positional.” Another limitation of our experiment is related to the use of changes in interpulse intervals to simulate the changes in the speed of the stimuli. The time delay between the pulses were kept constant for the constant speed sounds, while to simulate the accelerating and decelerating sounds, the interpulse intervals were decreasing and increasing, respectively. Hence, for these conditions (accelerating/decelerating sounds) the change in the pulse rate was covariant of the change in the rate of ITD. Hence, in order to successfully intercept these two kinds of sounds, in addition to ITD, listeners had additional cue to determine the instant of the temporal interception, though this was only the case in the accelerating/decelerating conditions, and not in the constant speed conditions. Therefore, it is possible to speculate that using moving sound sources in the free field as stimuli or simulating the sound sources by including other auditory spatial cues such as Doppler frequency shift, ILD and HRTF as well as employing continuous waves instead of pulse train would reveal different kinematic strategies used by the participants in the intercepting task, which was not unveiled by our stimuli.

## Conclusion

In this study, we tested whether and how the ITD of an arriving sound might be the main source of perceptual information for successfully intercepting a virtual moving sound source. The experimental findings revealed that in order to accomplish the task, one might need to vary different temporal and velocity parameters of the intercepting movements. The overall performance was more successful when subjects employed a time-to-contact (tau) coupling strategy and adjusted kinematic parameters such as duration, peak velocity and time to reach the peak velocity (speed coupling). This experiment emphasizes the need to better understand how humans pick up information in the auditory modality for the purposeful control of goal-directed behavior.

### Conflict of interest statement

The authors declare that the research was conducted in the absence of any commercial or financial relationships that could be construed as a potential conflict of interest.
